# Longitudinal studies on financial toxicity in cancer patients: a scoping review

**DOI:** 10.3389/fpubh.2026.1798871

**Published:** 2026-03-11

**Authors:** Ruiqi Lyu, Jianghua Wu

**Affiliations:** School of Nursing, Shandong First Medical University and Shandong Academy of Medical Sciences, Taian, Shandong, China

**Keywords:** cancer, financial toxicity, longitudinal study, temporal trend, oncology nursing, scoping review

## Abstract

**Objective:**

This scoping review focuses on financial toxicity in cancer patients, aiming to identify its measurement time points, assessment tools, developmental trends, and influencing factors. Based on existing evidence, this review reveals the occurrence trajectory and long-term impacts of financial toxicity among cancer patients. It can provide a reference for improving patients’ financial well-being in clinical practice and optimizing the medical security system at the health policy level and also points out key directions for future related research.

**Methods:**

This study strictly followed the methodology of scoping review. A systematic search was performed across the databases including PubMed, Web of Science, Embase, Cochrane Library, China National Knowledge Infrastructure (CNKI), Wan Fang Data, VIP, and Chinese Biomedical Literature Database (CBM). The search period covered from the inception of each database to November 8, 2025.

**Results:**

A total of 14 articles were included in this study. The common measurement time points were selected as before the initiation of treatment, 3 months, 6 months, and 12 months after the start of treatment. The research tools combined subjective scales with objective data measurement, and the Comprehensive Score for Financial Toxicity-Patient-Reported Outcome Measure (COST-PROM) was adopted in most studies. The changing trend of financial toxicity among breast cancer patients tended to be stable. The financial toxicity of colorectal cancer patients showed a continuous upward trend, while that of esophageal cancer patients presented a continuous downward trend. The financial toxicity of cervical cancer patients also demonstrated a declining trend, though the magnitude of change was smaller than that of esophageal cancer patients. A total of 25 influencing factors of financial toxicity in cancer patients were identified, involving four dimensions: sociodemographic factors (4), disease-related factors (4), economic-related factors (11), and psychosocial factors (6).

**Conclusion:**

The measurement time points of financial toxicity in cancer patients are mostly concentrated within one year after treatment, with insufficient research on long-term follow-up. The existing assessment tools lack applicability for long-term tracking and specificity for different cancer types, and objective indicators fail to effectively reflect patients’ economic status due to disparities in regional economic development levels. Patients with different types of cancer exhibit distinct developmental trends of financial toxicity, indicating population heterogeneity. Future research still needs to further explore the influencing factors and developmental trajectories of financial toxicity among different cancer types.

## 1 Introduction

With the continuous rise in the global incidence and mortality of cancer, cancer has become a major public health issue (1). Statistics show that the total number of cancer cases in China and the United States accounts for 24.2 and 11.9% of the global total, respectively (1, 2). Cancer imposes a heavy economic burden on patients and healthcare systems worldwide. Even with substantial investment in medical insurance, a considerable proportion of cancer patients still face severe financial hardship. From 2018 to 2025, China’s central fiscal investment in medical insurance exceeded 3 trillion yuan, yet 40% of patients still experienced financial toxicity (3, 4). However, this problem is not unique to developing countries; developed countries are also affected. Total healthcare expenditure on cancer treatment in the United States is projected to increase by an additional 44.9 billion USD by 2030 (5).

Financial toxicity refers to the adverse economic impact of cancer on patients and their families, and it has received increasing attention as an important patient-reported outcome (6). It includes direct costs (e.g., out-of-pocket expenses for treatment and medications) and indirect costs (e.g., income loss and reduced working hours), and is closely associated with treatment adherence, quality of life, and clinical outcomes (6–8).

Although a growing number of cancer studies have focused on financial toxicity, the vast majority adopted a cross-sectional design. In a cross-sectional study of 198 cancer patients, Perni et al. (9) found that more severe financial toxicity was associated with greater physical symptom burden and increased depression and anxiety. Similarly, a national cross-sectional survey of 664 Chinese breast cancer patients also identified the impacts of treatment modalities and demographic factors on financial toxicity (10). Overall, while these studies have demonstrated significant associations between financial toxicity and various psychological and physical symptoms, cross-sectional studies can only reveal associations at a single time point. They cannot clarify temporal order or causal direction, nor can they distinguish whether symptoms are risk factors or consequences of financial toxicity (11). More importantly, existing longitudinal evidence remains limited and fragmented. High heterogeneity exists across studies in measurement tools, follow-up duration, and assessment time points, making it difficult to determine the optimal screening timing and limiting the development of subsequent intervention studies and health-related policies.

Compared with cross-sectional studies, longitudinal studies can clarify the dynamic trajectory, long-term patterns, and predictive factors of financial toxicity throughout the cancer continuum. This scoping review examines the measurement time points, assessment tools, developmental trends, and influencing factors of financial toxicity among cancer patients in longitudinal studies. It aims to provide a reference for future research and standardized management of financial toxicity in cancer patients, and to support the development of evidence-based policies to reduce disparities and promote health equity.

## 2 Methods

A scoping review aims to examine the scope and nature of existing research on a topic or issue, determine the value of conducting a full systematic review, and identify gaps in current research (12). The methodology of a scoping review is based on the framework proposed by Arksey and O’Malley (13). We conducted this review in accordance with the Joanna Briggs Institute (JBI) Reviewer’s Manual approach (14), which includes the following stages: (a) identifying the review question; (b) inclusion and exclusion criteria; (c) search strategy; (d) evidence screening and selection; (e) data extraction; (f) data analysis; and (g) presentation of results. This scoping review was registered in the Open Science Framework (OSF) prior to the literature search, with the registration number 10.17605/OSF.IO/T795E. According to the scoping review methodology proposed by Peters et al. (14), quality assessment of the included evidence is generally not required. Therefore, no quality assessment was included in this review.

### 2.1 Identifying the review questions

The specific review questions are as follows: (1) What are the options for measurement time points in longitudinal studies on financial toxicity among cancer patients? (2) What are the options for assessment tools in longitudinal studies on financial toxicity among cancer patients? (3) What are the changing trends of financial toxicity among cancer patients? (4) What are the influencing factors of financial toxicity among cancer patients in longitudinal studies?

### 2.2 Eligibility criteria

The inclusion criteria were determined using the PICOS framework (Population, Intervention, Comparator, Outcome, Study design) (15). As the included studies were observational in nature, intervention and comparator components were not applicable.

Inclusion criteria:

(1) Study participants: Patients pathologically diagnosed with cancer, aged ≥ 18 years.
(2) Study design: Observational studies adopting a longitudinal research method.
(3) Outcome measures: Studies reporting the influencing factors of financial toxicity among cancer patients.
(4) Assessment standards: Studies using explicit assessment tools or diagnostic criteria for financial toxicity.

Exclusion criteria:

(1) Studies only presenting viewpoints without specific data or supporting materials;
(2) Studies not focusing on financial toxicity in cancer patients aged ≥18 years;
(3) Literature not written in English or Chinese;
(4) Reviews, dissertations, editorials, commentaries, and other forms of expert opinions.

### 2.3 Search strategy

A systematic search was conducted across the following databases: PubMed, Web of Science, Embase, Cochrane Library, China National Knowledge Infrastructure (CNKI), Wan Fang Data, VIP Chinese Science and Technology Journal Database, and Chinese Biomedical Literature Database (CBM). The search timeframe spanned from the inception of each database to November 8, 2025.

### 2.4 Screening and selection of evidence

The retrieved literature was imported into EndNote X9. First, duplicate studies were identified and removed. According to the inclusion and exclusion criteria, two trained researchers independently screened the titles and abstracts for initial eligibility, followed by a second screening through careful full-text review. Any disagreements were resolved through discussion with a third researcher. After confirming the included articles, the reference lists of the included studies were manually searched. Finally, all selected studies were checked by the reviewers. Data extracted from the included literature included: author, country, year of publication, cancer type, sample size, dropout rate, assessment tool and cutoff value, measurement time point, score, and influencing factors. The processes of study screening, data extraction, and analysis in this study were performed manually by researchers without the use of artificial intelligence (AI) tools.

### 2.5 Search outcome

**Figure 1:**
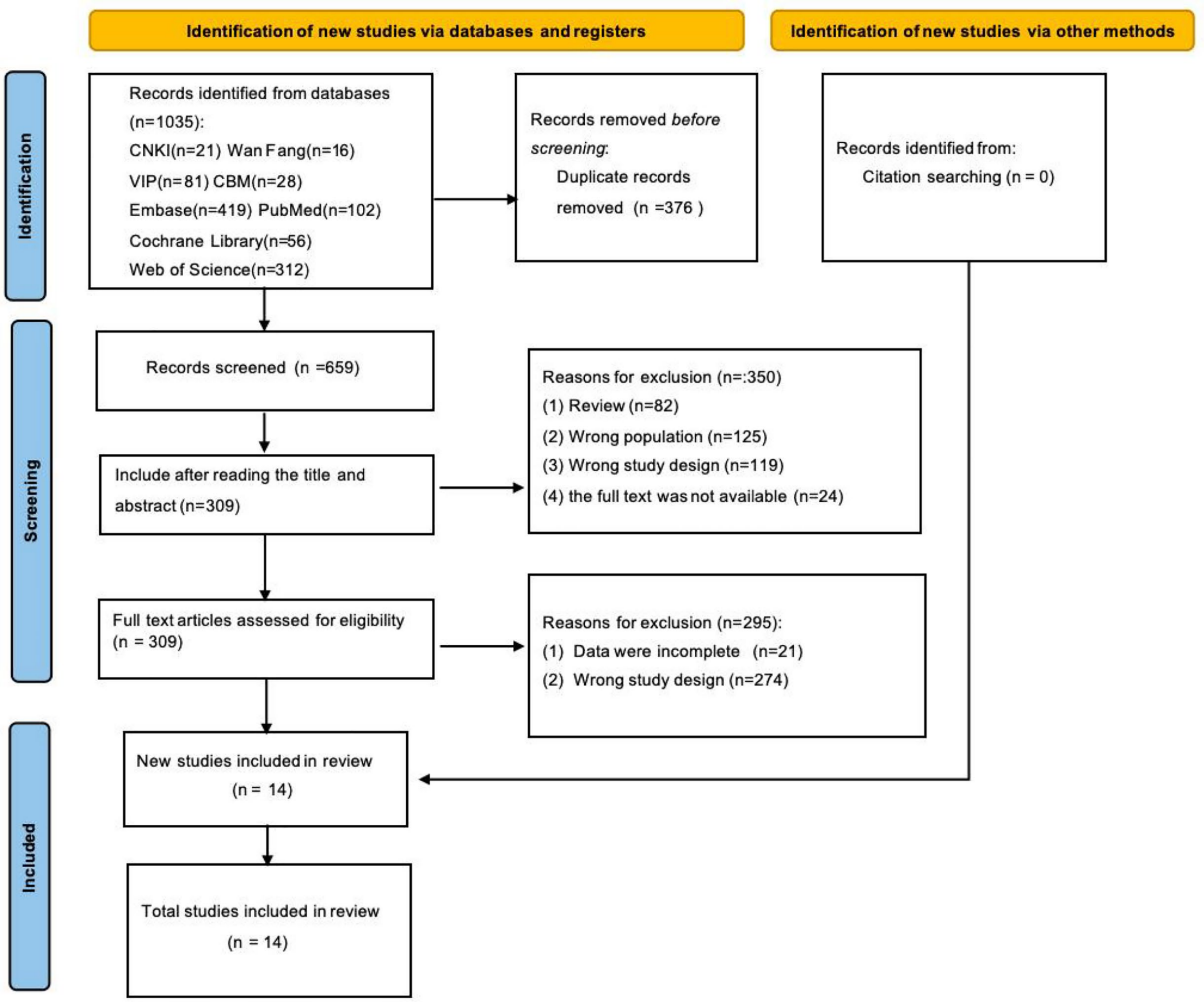
PRISMA flow diagram of study selection.

The initial search yielded a total of 1,035 articles. After removing 376 duplicate publications using EndNote X9, 659 records remained for the initial screening. According to the inclusion and exclusion criteria, 350 articles were excluded after screening the titles and abstracts, including 82 review articles, 125 articles with inappropriate study population, 119 articles with inappropriate study design, and 24 articles with unavailable full text. A total of 309 articles proceeded to full-text screening. During the full-text evaluation, 295 articles were further excluded, including 21 articles with incomplete data and 274 articles with inappropriate study design. After rigorous screening, a total of 14 articles were finally included in this scoping review (Figure 1).

## 3 Results

### 3.1 Literature search outcomes

A total of 1,035 articles were retrieved initially, and 14 studies were finally included after screening. Among the included articles, 6 were conducted in China (16–21), 6 in the United States (22–27), 1 in Germany (28), and 1 in Malaysia (29). The basic characteristics of the included studies are presented in detail in Table 1.

**Table 1:** Basic information of the included literature (*n* = 14).

Author	Country	Year	Tumor Type	Sample size	Attrition rate (%)	Assessment tool	Cut-off value	Measurement time point(s)	Score	Influencing factors
Li X. X. et al.	China	2025	Pancreatic cancer	340	23.8	COST-PROM	Score < 26 = positive	At admission, at discharge, 3 months after discharge, 6 months after discharge	——	(3) (4) (5) (6) (7)
Wu Y et al.	China	2025	Glioma	266	9.0	COST-PROM	Score < 26 = positive	Initial diagnosis; 3-month follow-up; 6-month follow-up	——	(3) (8)
Zheng Guimei et al.	China	2025	Cervical cancer	190	13.2	COST-PROM	Score < 26 = positive	After the first chemotherapy, 3 months later, and 6 months later	17.01 ± 8.90/15.15 ± 8.83/13.00 ± 8.47	(5) (9) (10)
Mark A Fiala et al.	USA	2025	Multiple myeloma	214	65	COST-PROM	Score < 26 = positive	After the diagnosis, one year later		(17) (23)
Westhofen T. et al.	German	2024	Prostatic cancer	397	—	The “Financial Toxicity Subscale” of EORTC QLQ-C30 (FTS)	FTS ≥ 17	The maximum follow-up period was 120 months, including before the operation, 3 months after the operation, and each year thereafter	——	(2) (13) (16) (24)
Kuang Yi et al.	China	2024	Breast cancer	447	15.4	COST-PROM	Score < 26 = positive	One week after the operation, three months after the operation, six months after the operation, and one year after the operation	23.47 ± 10.53/22.74 ± 11.37/24.01 ± 10.57/24.24 ± 10.49	(1) (2) (5) (9) (10) (11) (12) (13) (14)
Kircher S. et al.	USA	2024	Colorectal cancer	450	66.4	COST-PROM	Score < 26 = positive	Before the start of radiotherapy and chemotherapy, 3 months, 6 months, and 12 months	23.5 ± 11.9/25 ± 12.4/26.0 ± 12.1/28.6 ± 11.7	(3) (11) (15) (16)
Sadigh G. et al.	USA	2024	Colorectal cancer	451	51.9	COST-PROM	Score < 26 = positive	At the time of diagnosis, 3 months, 6 months, 12 months and 24 months	——	(17) (18) (19) (20)
Storandt M. H. et al.	USA	2023	Breast cancer	3,465	43.5	Projects that address the financial concerns raised	0–10 Score	At the time of diagnosis, 1 year later, 2 years later, 3 years later, 4 years later, 5 years later	2.76 ± 3.01/2.13 ± 2.8/2.04 ± 2.65/1.83 ± 2.56/1.78 ± 2.61/1.82 ± 2.65	(12) (17) (18)
Cheng Yuting et al.	China	2023	Brain Tumor	256 (pairs)	9.0	COST-PROM	Score < 26 = positive	Three days after diagnosis, six months	——	(8)
Friedes C. et al.	USA	2021	Lung cancer	215	47.9	COST-PROM	Score < 26 = positive	Within 5 weeks after the initial diagnosis or the start of cancer treatment, 6 months ±5 weeks		(5) (12) (21)
Liang M. I. et al.	USA	2021	Gynecological cancer	205	56.0	COST-PROM	Score < 26 = positive	Baseline (within 8 weeks after initiating the new systemic treatment regimen), 3 months and 6 months	23.6/25.1/25.6	(2) (16) (22)
Zhang Fagu et al.	China	2020	Esophagus cancer	100	——	The Self-perceived burden questionnaire includes an economic burden dimension	If the score is over 20, there is a sense of self-burden	One month, three months and six months after the operation	29.46 ± 7.45/19.55 ± 5.44/17.32 ± 4.77	(1)
Meram Azzani et al.	Malaysia	2016	Colorectal cancer	138	1.4	(1) Cost diary (2) Treatment cost Questionnaire and family Questionnaire (3) Inquiring about the subjective economic burden of patients and their families through a 5-point Likert score (4) Answering questions on financial coping strategies	——	At the time of diagnosis, six months after diagnosis, and one year after diagnosis	——	(13) (25) (20)

(1) Physical symptom burden (2) psychological symptom burden (3) self-efficacy (4) out-of-pocket expenses (5) monthly per capita household income (6) unplanned expenses (7) distance to hospital (8) family function (9) medical insurance (10) social support (11) place of residence (12) household savings (13) treatment method (14) subjective socioeconomic status (15) annual household income (16) impact on quality of life (17) age (18) gender (19) educational level (20) measurement time (21) ability to afford basic treatment (22) loss of wages, salaries or benefits (23) per capita household income (24) preoperative social function (25) tumor stage.

**Figure 2:**
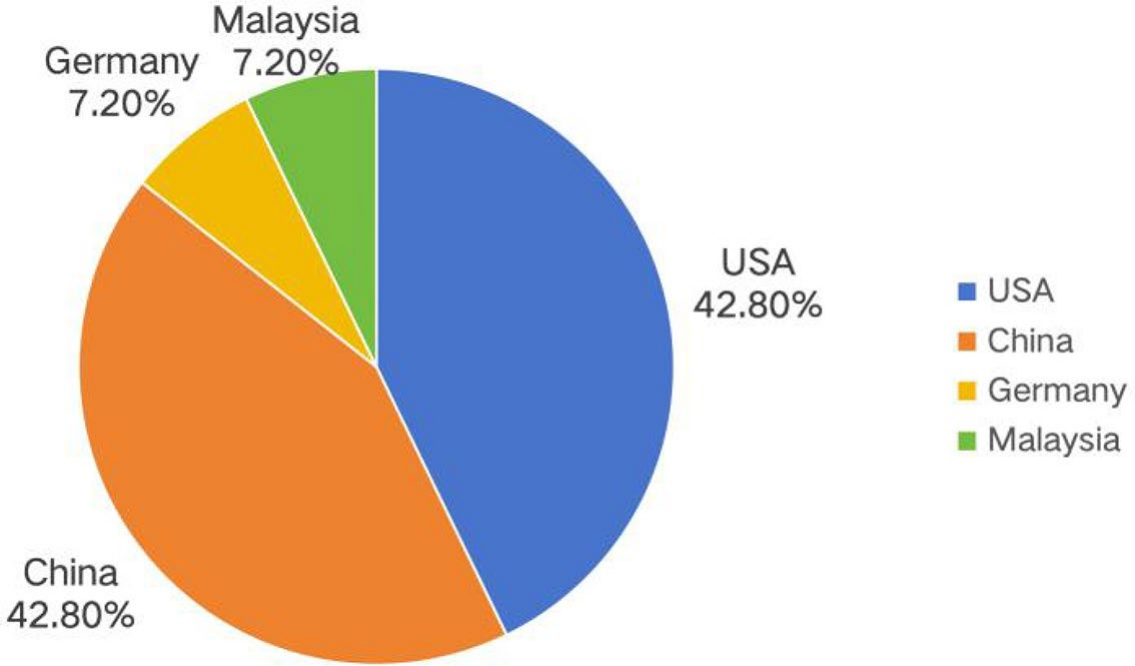
The pie chart illustrates the country distribution of the 14 studies included in this review.

The 14 included studies were distributed across 4 countries: the United States (*n* = 6, 42.9%) and China (*n* = 6, 42.9%) were the most predominant, while Germany and Malaysia each contributed 1 study (7.1% each). This highly concentrated geographic distribution indicates that the current evidence is mainly derived from North America and East Asia. Importantly, we identified a lack of relevant longitudinal studies in Africa and South America, which represents a substantial evidence gap in this field. This limitation suggests that our findings may not be generalizable to populations in these regions (Figure 2).

### 3.2 Measurement time points and trajectories of financial toxicity

**Figure 3:**
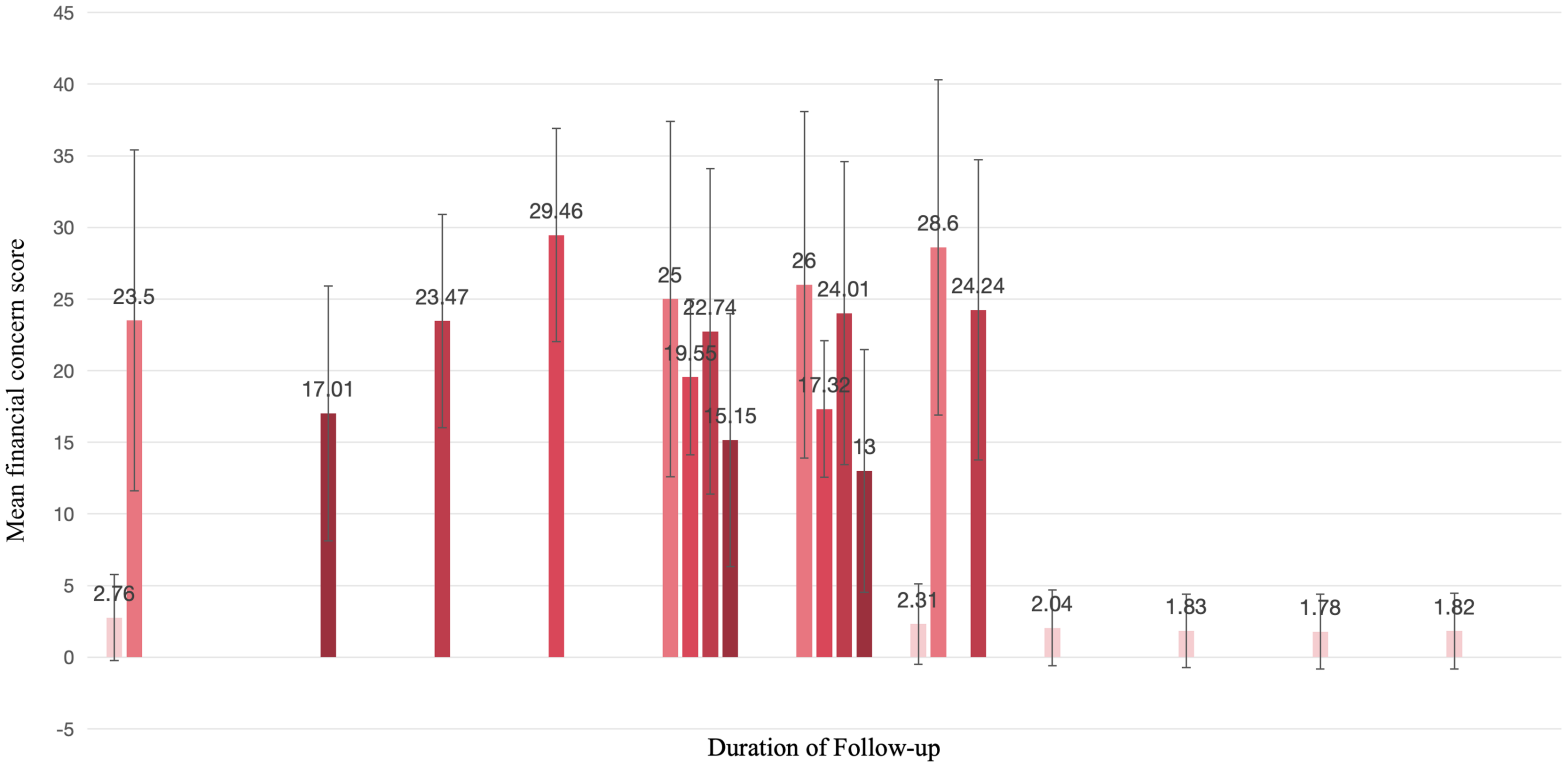
Selection of central tendency for longitudinal study time points in cancer patients.

All the included studies were longitudinal investigations on financial toxicity in cancer patients, with the number of measurements ranging from 2 to 6 times. Specifically, 4 studies adopted a 3-month interval between measurements (19, 21, 23, 25), while the remaining studies had fewer than 2 measurement time points. The longest follow-up duration was 5 years (24), and the shortest was 3 days after cancer diagnosis (17). Generally, after the first year of follow-up, researchers tended to set the interval of subsequent measurements as 1 year. The common measurement time points were selected as before the initiation of treatment, 3 months, 6 months, and 12 months after the start of treatment. The central tendency of measurement time point selection is illustrated in Figure 3.

**Figure 4:**
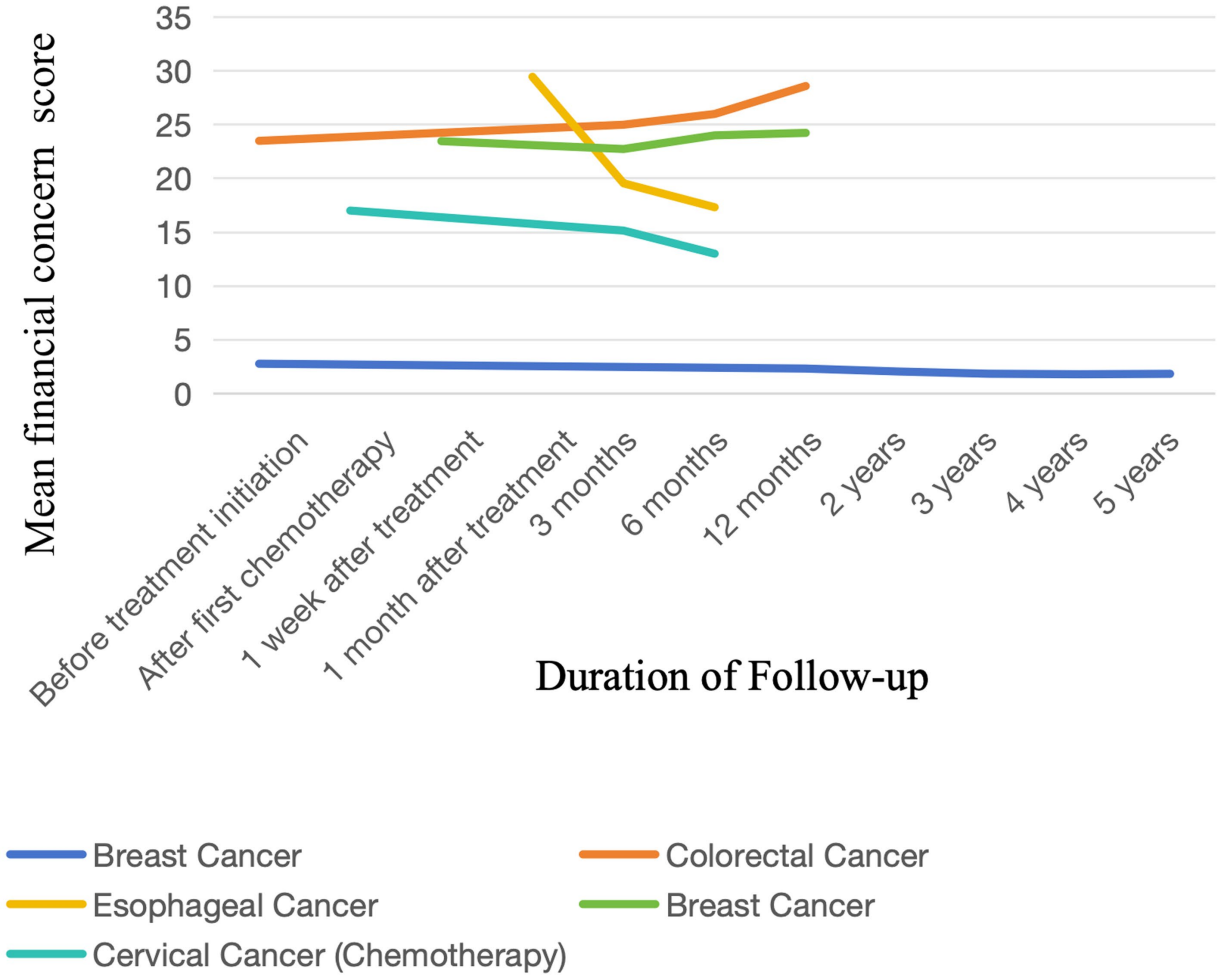
Longitudinal trajectories of economic toxicity in cancer patients.

Based on the 5 studies providing detailed data, the changing trend of financial toxicity among breast cancer patients tended to be stable with a small fluctuation range. The financial toxicity of colorectal cancer patients showed a continuous upward trend, with a greater increase after 6 months. For esophageal cancer patients, financial toxicity decreased continuously, with a particularly significant decline at 1 month and 6 months after treatment. The financial toxicity of cervical cancer patients also presented a downward trend, though the magnitude of change was smaller than that of esophageal cancer patients. Details are illustrated in Figure 4.

### 3.3 Assessment tools for financial toxicity in cancer patients

In the included studies, the follow-up investigation of financial toxicity in cancer patients was mainly conducted through two approaches: subjective and objective. The subjective approaches were mainly divided into three types: one was self-designed questions by researchers regarding patients’ economic status; another was the use of the Comprehensive Score for Financial Toxicity-Patient-Reported Outcome Measure (COST-PROM) questionnaire. Among the 14 included studies, 10 adopted the COST-PROM as the questionnaire for tracking patients’ financial toxicity. This questionnaire was first developed and published by American scholar DeSouza et al. (30) in 2013, and it is the first assessment tool designed for evaluating financial toxicity in cancer patients. As a self-reported scale for patients, it consists of 11 items, with a Cronbach’s *α* coefficient of 0.92 and a test–retest reliability of 0.80, indicating good reliability and validity (31). In the remaining studies, researchers recorded the development of patients’ financial toxicity through subscales within the scales. The objective approach mainly used cost diaries to record the changes in patients’ financial toxicity through objective data.

### 3.4 Influencing factors of financial toxicity in cancer patients

A total of 25 influencing factors were identified in the included studies, involving 4 aspects: sociodemographic factors (4), disease-related factors (4), economic-related factors (11), and psychosocial factors (6). Details are presented in Table 2.

**Table 2:** Influencing factors of longitudinal studies of economic toxicity in cancer patients.

Factor Category	Influencing factors	Number of literatures
Demographic factors	Place of residence	2
	Age	3
	Gender	2
	Educational level	1
Disease-related factors	Treatment method	3
	Measurement time	2
	Preoperative social function	1
	Tumor stage	1
Economic-related factors	Out-of-pocket expenses	1
	Monthly per capital household income	4
	Per capital household income	1
	Unplanned expenses	1
	Distance to hospital	1
	Medical insurance	2
	Household savings	3
	Subjective socioeconomic status	1
	Annual household income	1
	Ability to afford basic treatment	1
	Loss of wages, salaries or benefits	1
Psychosocial factors	Physical symptom burden	2
	Psychological symptom burden	3
	Self-efficacy	3
	Family function	2
	Social support	2
	Impact on quality of life	3

#### 3.4.1 Sociodemographic factors

Two studies (18, 25) indicated that living in regions with a lower level of economic development had a significant impact on the financial toxicity of cancer patients. Three studies (24, 26, 27) showed that age was an important influencing factor of financial toxicity. Two studies (24, 26) found that gender was also a key influencing factor, especially for males. One study (26) suggested that a higher educational level had a negative impact on financial toxicity.

#### 3.4.2 Disease-related factors

Three studies (18, 28, 29) demonstrated that the choice and quantity of treatment modalities had an important influence on the development of financial toxicity. Two studies (26, 29) found that the measurement time points selected in longitudinal follow-up studies played a crucial role in identifying the severity of financial toxicity. One study (28) revealed that patients’ social function before surgery had a significant impact on their financial toxicity after surgery. One study (29) indicated that tumor stage was an important influencing factor of financial toxicity.

#### 3.4.3 Economic-related factors

One study (20) indicated that patients’ out-of-pocket medical expenses exerted a significant impact on the development of financial toxicity. Four studies (18–20, 22) demonstrated that the per capita monthly household income played an important role in the development of financial toxicity among cancer patients. One study (27) showed that per capita household income was a crucial influencing factor of financial toxicity. One study (20) found that unexpected events experienced by patients had an impact on their financial toxicity. One study (20) suggested that the distance from patients’ residences to hospitals could affect their financial toxicity. Two studies (18, 19) revealed that different types of medical insurance had an influence on patients’ financial toxicity. Three studies (18, 22, 24) indicated that household savings were associated with patients’ financial toxicity. One study (18) showed that subjective social economic status had an impact on financial toxicity. One study (22) suggested that patients’ capacity to pay for treatment affected their financial toxicity. One study (23) found that the reduction in salaries, wages, and social welfare due to illness had a negative impact on financial toxicity. One study (25) discovered that the total annual household income was closely correlated with patients’ financial toxicity.

#### 3.4.4 Psychosocial factors

Two studies (16, 18) demonstrated that patients’ physical symptom burden had an important influence on financial toxicity. Three studies (18, 23, 28) indicated that patients’ psychological symptom burden affected their financial toxicity. Three studies (20, 21, 25) found that patients’ self-efficacy had a negative impact on financial toxicity. Two studies (17, 21) revealed that family function affected the subsequent development of patients’ financial toxicity. Two studies (18, 19) showed that social support played an important role in financial toxicity. Three studies (23, 25, 28) found that patients’ quality of life was also affected by financial toxicity.

## 4 Discussion

This scoping review included 14 articles and summarized the current evidence regarding financial toxicity among cancer patients. The aim of this study was to explore the long-term dynamic changes in financial toxicity, analyze relevant assessment tools, summarize influencing factors, and provide a basis for future intervention strategies.

Considerable heterogeneity was observed in the follow-up durations across included studies, with no unified standard, which limited the comparability across studies. Currently, no consistent guidelines define the appropriate follow-up period for financial toxicity. Most studies determined follow-up schedules based on clinical follow-up guidelines for specific cancer types. For example, guidelines recommend follow-up every 3 to 6 months for patients with colorectal cancer, with a colonoscopy required within one year after surgery (32). For patients with breast cancer, follow-up every 3 months is recommended during the first 3 years postoperatively (33). The lack of long-term longitudinal studies may be attributed to two main factors: first, financial toxicity is a sensitive issue involving patient privacy, often leading to high dropout rates; second, the study duration is closely related to investigators’ capacity and research objectives. Therefore, future studies should emphasize long-term follow-up to comprehensively reveal the dynamic trajectory of financial toxicity and develop targeted interventions based on the clinical characteristics of different cancers.

Financial toxicity showed a clear increasing trend among patients with colorectal cancer, which may be related to stoma construction following anal invasion. Both temporary and permanent stomas are associated with additional economic burden. For patients with temporary enterostomy, two surgeries (stoma construction and reversal) result in substantially higher medical costs than those for most other cancer patients (34). Furthermore, long-term stoma care further increases financial toxicity (35). Notably, in this review, financial toxicity decreased significantly among patients with esophageal cancer and cervical cancer undergoing chemotherapy, while it remained relatively stable among breast cancer patients. This discrepancy may be explained by two factors: first, within 12 months after diagnosis, most patients become familiar with medical insurance policies, which alleviates financial anxiety caused by cost uncertainty (25). Second, patients with esophageal cancer and cervical cancer do not require stoma care, which may reduce their financial burden. However, comparative studies on financial toxicity across different cancer types remain limited, weakening the generalizability of these findings. Further research with larger datasets is needed to verify whether patients with colorectal cancer experience more severe financial burden than those with other cancers.

Current assessment tools present notable limitations that compromise the reliability of study conclusions. Although the COST questionnaire is widely used, its 7-day recall period is insufficient to capture dynamic changes in financial toxicity, and its non-cancer-specific design may introduce bias. In addition, self-designed questionnaires and subscales lack standardized criteria, and their reliability and validity are often unproven. For objective measurement, cost diaries rely on self-reporting, which limits data accuracy and timeliness. Moreover, no universal standard exists for currency conversion in cross-national studies. Future tools should integrate subjective and objective indicators, be cancer-specific, and account for regional economic development levels to enable standardized assessment. Such tools would improve comparability and representativeness, thereby supporting more efficient allocation of medical resources and better identification of patients at high risk of financial toxicity.

In this study, financial toxicity among cancer patients was influenced by multiple factors, which were closely associated with national economic conditions, social infrastructure, and healthcare security systems. We observed that financial toxicity also exists in developed countries with well-established public healthcare systems. Systematic reviews have indicated that even in countries with universal public health insurance, material hardship persists due to insufficient public financial coverage for rising out-of-pocket costs and income loss related to cancer diagnosis (36). However, the severity of financial toxicity is generally lower in developed countries than in developing countries (37). One study suggested that barriers to healthcare may be absolute for older adults individuals in rural areas of sub-Saharan Africa (9). Persistent inequalities driven by political, social, and institutional factors may affect cancer survivors. Notably, financial toxicity includes not only direct medical costs but also indirect and non-medical costs. In developing countries, in particular, many patients seek medical care in urban tertiary hospitals, and long-distance transportation and temporary accommodation can become substantial burdens. Within the same country, the regional economic level significantly affects financial toxicity, with higher risks in less developed areas. Data show that 50% of rural patients experienced varying degrees of financial toxicity, compared with only 39% of urban patients, due to challenges such as limited reimbursement channels, low income, insufficient staffing, and additional costs caused by geographical barriers (38, 39). Importantly, the economic consequences of cancer extend beyond patients to their families. Measuring only out-of-pocket expenses is insufficient to understand the full financial burden of cancer (40). Focusing solely on out-of-pocket costs may be misleading and fail to identify those most in need. Future research should pay more attention to developing and economically disadvantaged regions and compare whether they experience different types or degrees of financial difficulty compared with developed regions. In addition, further investigations are needed to clarify the real indirect burden borne by family members and friends. These findings have implications for the assessment, intervention, and support for families’ financial concerns, which should ideally be addressed alongside patient care.

Longitudinal studies allow more accurate identification of the temporal sequence of influencing factors across different disease stages, supporting early intervention. Existing evidence indicates that financial toxicity exacerbates physical symptom burden, patient-provider communication, and trust, further complicating symptom management (9, 41). The lack of long-term data prevents researchers from capturing the cumulative effects and critical turning points of financial toxicity. For instance, patients may encounter episodic payment difficulties during adjuvant therapy due to cumulative out-of-pocket costs. Without timely identification, this may lead to delayed treatment, dose reduction, or unplanned treatment discontinuation, increasing the risk of disease progression and overall medical costs. Furthermore, financially distressed patients may hide their difficulties due to stigma or abandon cost-effective maintenance therapy because of uncertain prognosis. Therefore, future studies should implement long-term follow-up covering the entire treatment and survivorship period to clarify the dynamic evolution of financial toxicity and establish real-time early warning systems to avoid irreversible health damage caused by delayed intervention.

## 5 Limitations

First, as all included studies were conducted in North America, East Asia, and parts of Europe, the generalizability of the findings is limited, with underrepresentation of populations in Africa and South America. Future research should prioritize longitudinal investigations in these regions to address this critical evidence gap. Second, this review only included Chinese and English literature, which may have resulted in incomplete retrieval of relevant studies. Finally, due to the high heterogeneity of the available data, conclusions regarding the influencing factors of financial toxicity among cancer patients may vary, and follow-up with additional relevant studies is warranted for further validation and analysis.

## 6 Conclusion

This review included a limited number of studies with relatively short follow-up durations and uneven distribution across cancer types; thus, conclusions regarding differences in financial toxicity among different cancers need to be verified by more high-quality research. At present, the tools used to evaluate the financial toxicity of cancer patients lack specificity for different cancer types, and there is heterogeneity in assessment methods for long-term follow-up evaluations. Although existing studies have mentioned the use of objective data for assessment, due to the differences in economic development levels across different regions, it is difficult to form a unified evaluation formula to accurately reflect the development of patients’ financial toxicity. Therefore, future research should focus on advancing three aspects of work: first, further develop long-term follow-up financial toxicity assessment tools for different cancer types, and construct an evaluation system combining subjective and objective approaches; second, carry out targeted research and intervention focusing on the key time nodes of financial toxicity development in different cancer types, to accurately alleviate patients’ economic burden; third, strengthen comparative research on the heterogeneity of financial toxicity development among different cancer types, to provide a solid basis for the formulation of personalized intervention programs in the future.

## References

[1] Wu Y He S Cao M Teng Y Li Q Tan N . Comparative analysis of cancer statistics in China and the United States in 2024. Chin Med J. (2024) 137:3093–100. doi: 10.1097/CM9.0000000000003442, 39654104 PMC11706596

[2] Filho AM Laversanne M Ferlay J Colombet M Piñeros M Znaor A . The GLOBOCAN 2022 cancer estimates: data sources, methods, and a snapshot of the cancer burden worldwide. Int J Cancer. (2025) 156:1336–1346. doi: 10.1002/ijc.35278, 39688499

[3] Zafar SY Peppercorn JM Schrag D Taylor DH Goetzinger AM Zhong X . The financial toxicity of cancer treatment: a pilot study assessing out-of-pocket expenses and the insured cancer patient's experience. Oncologist. (2013) 18:381–390. doi: 10.1634/theoncologist.2012-0279, 23442307 PMC3639525

[4] Wang S Wang J Kang H Zeng L Liu G Qiu Y . Assessment of the prevalence and related factors of financial toxicity in cancer patients based on the COST scale: a systematic review and meta-analysis. Eur J Oncol Nurs. (2024) 68:102489. doi: 10.1016/j.ejon.2023.102489, 38118267

[5] Sloan FA Yashkin AP Akushevich I Inman BA. Longitudinal patterns of cost and utilization of medicare beneficiaries with bladder cancer. Urol Oncol. (2020) 38:39.e11–9. doi: 10.1016/j.urolonc.2019.10.016, 31761612

[6] Abrams HR Sienna D Huang CX Johnson SF Nayak RK Zahner GJ . Financial toxicity in cancer care: origins, impact, and solutions. Transl Behav Med. (2021) 11:2043–2054. doi: 10.1093/tbm/ibab091 34850932

[7] Ehlers M Bjurlin M Gore J Pruthi R Narang G Tan R . A national cross-sectional survey of financial toxicity among bladder cancer patients. Urol Oncol. (2021) 39: 76.e1–76.e7. doi: 10.1016/j.urolonc.2020.09.030 33268274

[8] Ting CY Teh GC Yu KL Alias H Tan HM Wong LP. Financial toxicity and its associations with health-related quality of life among urologic cancer patients in an upper middle-income country. Support Care Cancer. (2020) 28:1703–15. doi: 10.1007/s00520-019-04975-y, 31292755

[9] Perni S Azoba C Gorton E Park ER Chabner BA Moy B . Financial toxicity, symptom burden, illness perceptions, and communication confidence in cancer clinical trial participants. JCO Oncol Pract. (2022) 18:e1427–37. doi: 10.1200/op.21.00697, 35666957

[10] Liu M Hu L Han X Cao M Sun J Liu Y. Financial toxicity in female patients with breast cancer: a national cross-sectional study in China. Support Care Cancer. (2022) 30:8231–40. doi: 10.1007/s00520-022-07264-3, 35819521 PMC9512750

[11] Carrera PM Curigliano G Santini D Sharp L Chan RJ Pisu M . ESMO expert consensus statements on the screening and management of financial toxicity in patients with cancer. ESMO Open. (2024) 9:102992. doi: 10.1016/j.esmoop.2024.102992, 38626634 PMC11033153

[12] Peters MD Godfrey CM Khalil H Peters MDJ McInerney P Parker D . Guidance for conducting systematic scoping reviews. Int J Evid Based Healthc. (2015) 13:141–6. doi: 10.1097/xeb.0000000000000050, 26134548

[13] Arksey H O"Malley L. Scoping studies: towards a methodological framework. Int J Soc Res Methodol. (2005) 8:19–32. doi: 10.1080/1364557032000119616

[14] Peters MDJ Marnie C Tricco AC Pollock D Munn Z Alexander L . Updated methodological guidance for the conduct of scoping reviews. JBI Evid Implement. (2021) 19:3–10. doi: 10.1097/xeb.0000000000000277, 33570328

[15] Lockwood C Dos Santos KB Pap R. Practical guidance for knowledge synthesis: scoping review methods. Asian Nurs Res (Korean Soc Nurs Sci). (2019) 13:287–94. doi: 10.1016/j.anr.2019.11.002, 31756513

[16] Zhang FZ Yu H . A longitudinal study on the quality of life and self-perceived burden of patients during the recovery period after radical esophageal cancer surgery [In Chinese]. Integrated Traditional Chinese Western Med Nursing. (2020) 6:109–113.

[17] Cheng YT Zhou DY Zhang C . A longitudinal subject-object model study on the family function and economic toxicity of patients with brain tumors and their caregivers [In Chinese]. J Nurs Sci. (2023) 38:15–19.

[18] Kuang Y Qiu JJ Tang LC Liu Y Guo SJ Chen T . A longitudinal study on the economic toxicity level and influencing factors of patients after breast cancer surgery [In Chinese]. Nurse Continuing Educ. J. (2024) 39:2023–2030. doi: 10.16821/j.cnki.hsjx.2024.19.002

[19] Zheng GM Mao BD Qian SL. Trajectory of financial toxicity and its influencing factors in patients with cervical cancer undergoing chemotherapy. Psychological Monthly.(2025) 20:20–23. doi: 10.19738/j.cnki.psy.2025.12.005

[20] Xiaoxuan L Shuo Z Shanshan L Mengxia Y Tianying Y Yuxin S . Predictors of financial toxicity trajectories in patients with pancreatic cancer: a latent class growth analysis. Cancer Med. (2025) 14:e70875. doi: 10.1002/cam4.70875 PMC12050404 40320835

[21] Wu Y Wu YH Zhou BT Zhou B Sun Y Dai R. Trajectories of family functioning and financial toxicity in patients with glioma: a longitudinal study. Front Public Health. (2025) 13:1573000. doi: 10.3389/fpubh.2025.1573000, 40756411 PMC12313587

[22] Friedes C Hazell SZ Fu W Hu C Voong RK Lee B . Longitudinal trends of financial toxicity in patients with lung Cancer: a prospective cohort study. Jco Oncology Practice. (2021) 17:e1094–E1109. doi: 10.1200/op.20.00721, 33555936

[23] Liang MI Summerlin SS Blanchard CT Boitano TKL Huh WK Bhatia S . Measuring financial distress and quality of life over time in patients with gynecologic Cancer—making the case to screen early in the treatment course. JCO Oncology Practice. (2021) 17:E1576–83. doi: 10.1200/OP.20.00907, 33596114 PMC8791820

[24] Storandt MH Durani U Stan D Larson N Loprinzi C Couch F . Financial hardship in breast cancer survivors: a prospective analysis of change in financial concerns over time. Support Care Cancer. (2023) 31:62. doi: 10.1007/s00520-022-07493-6, 36534173

[25] Kircher S Duan F An N Gareen IF Sicks JD Sadigh G . Patient-reported financial burden of treatment for Colon or rectal Cancer. JAMA Netw Open. (2024) 7:e2350844. doi: 10.1001/jamanetworkopen.2023.50844, 38194233 PMC10777253

[26] Sadigh G Duan FH An N Duan F Gareen ID Sicks JR . Financial hardship among patients with early-stage colorectal cancer. JAMA Netw Open. (2024) 7:e2431967. doi: 10.1001/jamanetworkopen.2024.31967, 39287948 PMC11409151

[27] Fiala MA Lepisto E Amadi-Mgbenka C Schulman J Mulligan G Cho HJ. Financial toxicity and satisfaction with cancer treatment among patients with multiple myeloma: an analysis of the MMRF'S CureCloud initiative. Cancer Res. (2025) 85:e254–e260. doi: 10.1016/j.clml.2025.10.007 41184167

[28] Westhofen T Buchner A Eismann L Rodler S Becker A Stief CG . De novo financial toxicity in patients with prostate cancer following radical prostatectomy and its impact on health-related quality of life. J Clin Oncol. (2024) 42:318. doi: 10.1200/JCO.2024.42.4_suppl.318

[29] Azzani M Roslani AC Su TT. Financial burden of colorectal cancer treatment among patients and their families in a middle-income country. Support Care Cancer. (2016) 24:4423–32. doi: 10.1007/s00520-016-3283-2, 27225528

[30] de Souza JA Yap BJ Hlubocky FJ Wroblewski K Ratain MJ Cella D . The development of a financial toxicity patient-reported outcome in cancer: the COST measure. Cancer. (2014) 120:3245–3253. doi: 10.1002/cncr.28814 24954526

[31] de Souza JA Yap BJ Wroblewski K Blinder V Araújo FS Hlubocky FJ . Measuring financial toxicity as a clinically relevant patient-reported outcome: the validation of the COmprehensive score for financial toxicity (COST). Cancer. (2017) 123:476–84. doi: 10.1002/cncr.30369, 27716900 PMC5298039

[32] Chinese College of Surgeons, Chinese Medical Doctor Association, Chinese Society Gastrointestinal Surgery, Chinese Society of Surgery, Chinese Medical Association, Chinese Society of Colorectal Surgery et al. Chinese guidelines for diagnosis and comprehensive treatment of colorectal liver metastases (2020 edition) [In Chinese]. Chin J Clin Hepatol. (2021) 37:543–53.

[33] Tianyuan L Zhongjian Z Zhenggui D. Interpretation of clinical practice guidelines for the diagnosis, treatment and follow-up of early breast cancer. Chin J Thoracic Cardiovas Surg Clinic Stud. (2025) 32:1072–8.

[34] Wu QZ Liu JJ Chen GL Wei YH Li H. Influencing factors associated with peristomal dermatitis risk in patients with permanent end colostomy for rectal cancer [In Chinese]. Chin J Health Care Med. (2022)24:162–164.

[35] Yao Y Zhang S Yu Q Zhao X Zhang X. The financial toxicity experience of patients with colorectal Cancer during chemotherapy: a qualitative study. Curr Oncol. (2024) 32:23. doi: 10.3390/curroncol32010023, 39851939 PMC11764152

[36] Lee S Olvera RG Shiu-Yee K Rush LJ Tarver WL Blevins T . Short-term and long-term financial toxicity from breast cancer treatment: a qualitative study. Support Care Cancer. (2023) 32:24. doi: 10.1007/s00520-023-08199-z, 38095729

[37] Çeli KY Çeli KS Sarıköse S Arslan HN. Evaluation of financial toxicity and associated factors in female patients with breast cancer: a systematic review and meta-analysis. Support Care Cancer. (2023) 31:691. doi: 10.1007/s00520-023-08172-w 37953376

[38] Zahnd WE Davis MM Rotter JS Vanderpool RC Perry CK Shannon J . Rural-urban differences in financial burden among cancer survivors: an analysis of a nationally representative survey. Support Care Cancer. (2019) 27:4779–86. doi: 10.1007/s00520-019-04742-z, 30972645 PMC6786922

[39] Deshmukh Y Rose ML Yen RW Jones ST Kapadia NS. Rural cancer financial toxicity screening. Adv Radiat Oncol. (2025) 10:101910. doi: 10.1016/j.adro.2025.101910, 41333179 PMC12666702

[40] Fitch MI Sharp L Hanly P Longo CJ. Experiencing financial toxicity associated with cancer in publicly funded healthcare systems: a systematic review of qualitative studies. J Cancer Surviv. (2022) 16:314–28. doi: 10.1007/s11764-021-01025-7, 33723742

[41] Kuang Y Qi X Qiu JJ Qiu J Liu Y Guo S . Unraveling the vicious cycle: longitudinal analysis between financial toxicity and symptom burden in women with breast cancer. Support Care Cancer. (2025) 33:829. doi: 10.1007/s00520-025-09873-0, 40892242

