# Supplementary material for: Longitudinal studies on financial toxicity in cancer patients: a scoping review
Source: Front Public Health. 2026 Mar 11;14:1798871. doi: 10.3389/fpubh.2026.1798871 (PMC13013514; doi:10.3389/fpubh.2026.1798871)
Supplement: Supplementary file 1 [file Table_1.DOCX]

Date：2025-11-08

Database：Pubmed

| Search | Query | Results |
| --- | --- | --- |
| #1 | Search: "Neoplasms"[Mesh] | 4,172,586 |
| #2 | Search: (Tumors[Title/Abstract]) OR (Neoplasia[Title/Abstract]) OR (Neoplasias[Title/Abstract]) OR (Neoplasm[Title/Abstract]) OR (Tumor[Title/Abstract]) OR (Cancer[Title/Abstract]) OR (Cancers[Title/Abstract]) OR (Malignant Neoplasm[Title/Abstract]) OR (Malignancy[Title/Abstract]) OR (Malignancies[Title/Abstract]) OR (Malignant Neoplasms[Title/Abstract]) OR (Neoplasm, Malignant[Title/Abstract]) OR (Neoplasms, Malignant[Title/Abstract]) OR (Benign Neoplasms[Title/Abstract]) OR (Neoplasms, Benign[Title/Abstract]) OR (Neoplasm, Benign[Title/Abstract]) OR (Benign Neoplasm[Title/Abstract]) | 3,898,658 |
| **#3** | Search: (Neoplasms[MeSH Terms]) OR ((Tumors[Title/Abstract]) OR (Neoplasia[Title/Abstract]) OR (Neoplasias[Title/Abstract]) OR (Neoplasm[Title/Abstract]) OR (Tumor[Title/Abstract]) OR (Cancer[Title/Abstract]) OR (Cancers[Title/Abstract]) OR (Malignant Neoplasm[Title/Abstract]) OR (Malignancy[Title/Abstract]) OR (Malignancies[Title/Abstract]) OR (Malignant Neoplasms[Title/Abstract]) OR (Neoplasm, Malignant[Title/Abstract]) OR (Neoplasms, Malignant[Title/Abstract]) OR (Benign Neoplasms[Title/Abstract]) OR (Neoplasms, Benign[Title/Abstract]) OR (Neoplasm, Benign[Title/Abstract]) OR (Benign Neoplasm[Title/Abstract])) | 5,437,960 |
| #4 | Search: "Financial Stress"[Mesh] | 1,618 |
| #5 | Search: (Financial Stresses[Title/Abstract]) OR (Stress, Financial[Title/Abstract]) OR (Economic Burden[Title/Abstract]) OR (Burden, Economic[Title/Abstract]) OR (Economic Burdens[Title/Abstract]) OR (Financial Burden[Title/Abstract]) OR (Burden, Financial[Title/Abstract]) OR (Financial Burdens[Title/Abstract]) OR (Financial Toxicity[Title/Abstract]) OR (Financial Toxicities[Title/Abstract]) OR (Toxicity, Financial[Title/Abstract]) OR (Financial Challenges[Title/Abstract]) OR (Challenge, Financial[Title/Abstract]) OR (Financial Challenge[Title/Abstract]) OR (Financial Pressures[Title/Abstract]) OR (Financial Pressure[Title/Abstract]) OR (Pressure, Financial[Title/Abstract]) OR (Financial Strain[Title/Abstract]) OR (Financial Strains[Title/Abstract]) OR (Strain, Financial[Title/Abstract]) OR (Insufficient Financial Resources[Title/Abstract]) OR (Financial Resource, Insufficient[Title/Abstract]) OR (Insufficient Financial Resource[Title/Abstract]) OR (Resource, Insufficient Financial[Title/Abstract]) OR (Financial Resource Strain[Title/Abstract]) OR (Financial Resource Strains[Title/Abstract]) OR (Resource Strain, Financial[Title/Abstract]) OR (Strain, Financial Resource[Title/Abstract]) OR (Socioeconomic Adversity[Title/Abstract]) OR (Adversity, Socioeconomic[Title/Abstract]) OR (Socioeconomic Adversities[Title/Abstract]) OR (Financial Hardship[Title/Abstract]) OR (Financial Hardships[Title/Abstract]) OR (Hardship, Financial[Title/Abstract]) OR (Economic Hardship[Title/Abstract]) OR (Economic Hardships[Title/Abstract]) OR (Hardship, Economic[Title/Abstract]) | 41,878 |
| **#6** | Search: ((Financial Stresses[Title/Abstract]) OR (Stress, Financial[Title/Abstract]) OR (Economic Burden[Title/Abstract]) OR (Burden, Economic[Title/Abstract]) OR (Economic Burdens[Title/Abstract]) OR (Financial Burden[Title/Abstract]) OR (Burden, Financial[Title/Abstract]) OR (Financial Burdens[Title/Abstract]) OR (Financial Toxicity[Title/Abstract]) OR (Financial Toxicities[Title/Abstract]) OR (Toxicity, Financial[Title/Abstract]) OR (Financial Challenges[Title/Abstract]) OR (Challenge, Financial[Title/Abstract]) OR (Financial Challenge[Title/Abstract]) OR (Financial Pressures[Title/Abstract]) OR (Financial Pressure[Title/Abstract]) OR (Pressure, Financial[Title/Abstract]) OR (Financial Strain[Title/Abstract]) OR (Financial Strains[Title/Abstract]) OR (Strain, Financial[Title/Abstract]) OR (Insufficient Financial Resources[Title/Abstract]) OR (Financial Resource, Insufficient[Title/Abstract]) OR (Insufficient Financial Resource[Title/Abstract]) OR (Resource, Insufficient Financial[Title/Abstract]) OR (Financial Resource Strain[Title/Abstract]) OR (Financial Resource Strains[Title/Abstract]) OR (Resource Strain, Financial[Title/Abstract]) OR (Strain, Financial Resource[Title/Abstract]) OR (Socioeconomic Adversity[Title/Abstract]) OR (Adversity, Socioeconomic[Title/Abstract]) OR (Socioeconomic Adversities[Title/Abstract]) OR (Financial Hardship[Title/Abstract]) OR (Financial Hardships[Title/Abstract]) OR (Hardship, Financial[Title/Abstract]) OR (Economic Hardship[Title/Abstract]) OR (Economic Hardships[Title/Abstract]) OR (Hardship, Economic[Title/Abstract]) AND (1000/1/1:2025/4/30[pdat])) OR ("Financial Stress"[Mesh] | 42,085 |
| #8 | Search: "Longitudinal Studies"[Mesh] | 190,766 |
| #9 | Search: (((((((((((((((((((((((((((((((Longitudinal Study[Title/Abstract]) OR (Studies, Longitudinal[Title/Abstract])) OR (Study, Longitudinal[Title/Abstract])) OR (Longitudinal Survey[Title/Abstract])) OR (Longitudinal Surveys[Title/Abstract])) OR (Survey, Longitudinal[Title/Abstract])) OR (Surveys, Longitudinal[Title/Abstract])) OR (Bogalusa Heart Study[Title/Abstract])) OR (Bogalusa Heart Studies[Title/Abstract])) OR (Heart Studies, Bogalusa[Title/Abstract])) OR (Heart Study, Bogalusa[Title/Abstract])) OR (Studies, Bogalusa Heart[Title/Abstract])) OR (Study, Bogalusa Heart[Title/Abstract])) OR (California Teachers Study[Title/Abstract])) OR (California Teachers Studies[Title/Abstract])) OR (Studies, California Teachers[Title/Abstract])) OR (Study, California Teachers[Title/Abstract])) OR (Teachers Studies, California[Title/Abstract])) OR (Teachers Study, California[Title/Abstract])) OR (Jackson Heart Study[Title/Abstract])) OR (Heart Studies, Jackson[Title/Abstract])) OR (Heart Study, Jackson[Title/Abstract])) OR (Jackson Heart Studies[Title/Abstract])) OR (Studies, Jackson Heart[Title/Abstract])) OR (Tuskegee Syphilis Study[Title/Abstract])) OR (Syphilis Studies, Tuskegee[Title/Abstract])) OR (Syphilis Study, Tuskegee[Title/Abstract])) OR (Tuskegee Syphilis Studies[Title/Abstract])) OR (Framingham Heart Study[Title/Abstract])) OR (Framingham Heart Studies[Title/Abstract])) OR (Heart Studies, Framingham[Title/Abstract])) OR (Heart Study, Framingham[Title/Abstract]) | 105,467 |
| **#10** | Search: ((((((((((((((((((((((((((((((((Longitudinal Study[Title/Abstract]) OR (Studies, Longitudinal[Title/Abstract])) OR (Study, Longitudinal[Title/Abstract])) OR (Longitudinal Survey[Title/Abstract])) OR (Longitudinal Surveys[Title/Abstract])) OR (Survey, Longitudinal[Title/Abstract])) OR (Surveys, Longitudinal[Title/Abstract])) OR (Bogalusa Heart Study[Title/Abstract])) OR (Bogalusa Heart Studies[Title/Abstract])) OR (Heart Studies, Bogalusa[Title/Abstract])) OR (Heart Study, Bogalusa[Title/Abstract])) OR (Studies, Bogalusa Heart[Title/Abstract])) OR (Study, Bogalusa Heart[Title/Abstract])) OR (California Teachers Study[Title/Abstract])) OR (California Teachers Studies[Title/Abstract])) OR (Studies, California Teachers[Title/Abstract])) OR (Study, California Teachers[Title/Abstract])) OR (Teachers Studies, California[Title/Abstract])) OR (Teachers Study, California[Title/Abstract])) OR (Jackson Heart Study[Title/Abstract])) OR (Heart Studies, Jackson[Title/Abstract])) OR (Heart Study, Jackson[Title/Abstract])) OR (Jackson Heart Studies[Title/Abstract])) OR (Studies, Jackson Heart[Title/Abstract])) OR (Tuskegee Syphilis Study[Title/Abstract])) OR (Syphilis Studies, Tuskegee[Title/Abstract])) OR (Syphilis Study, Tuskegee[Title/Abstract])) OR (Tuskegee Syphilis Studies[Title/Abstract])) OR (Framingham Heart Study[Title/Abstract])) OR (Framingham Heart Studies[Title/Abstract])) OR (Heart Studies, Framingham[Title/Abstract])) OR (Heart Study, Framingham[Title/Abstract])) OR ("Longitudinal Studies"[Mesh]) | 230,159 |
| #11 | #3 AND #6 AND #10 | 102 |

Database: Web of Science

Publication Date：1985-01-01 to 2025-04-30

| #1 | Ti=(Neoplasms OR Tumors OR Neoplasia OR Neoplasias OR Neoplasm OR Tumor OR Cancer OR Cancers OR Malignant Neoplasm OR Malignancy OR Malignancies OR Malignant Neoplasms OR Neoplasm, Malignant OR Neoplasms, Malignant OR Benign Neoplasms OR Neoplasms, Benign OR Neoplasm, Benign OR Benign Neoplasm) | 3,187,386 |
| --- | --- | --- |
| #2 | TI=(Financial Stress OR Financial Stresses OR Stress, Financial OR Economic Burden OR Burden, Economic OR Economic Burdens OR Financial Burden OR Burden, Financial OR Financial Burdens OR Financial Toxicity OR Financial Toxicities OR Toxicity, Financial OR Financial Challenges OR Challenge, Financial OR Financial Challenge OR Financial Pressures OR Financial Pressure OR Pressure, Financial OR Financial Strain OR Financial Strains OR Strain, Financial OR Insufficient Financial Resources OR Financial Resource, Insufficient OR Insufficient Financial Resource OR Resource, Insufficient Financial OR Financial Resource Strain OR Financial Resource Strains OR Resource Strain, Financial OR Strain, Financial Resource OR Socioeconomic Adversity OR Adversity, Socioeconomic OR Socioeconomic Adversities OR Financial Hardship OR Financial Hardships OR Hardship, Financial OR Economic Hardship OR Economic Hardships OR Hardship, Economic) | 133,359 |
| #3 | TI=(Longitudinal Studies OR Longitudinal Study OR Studies, Longitudinal OR Study, Longitudinal OR Longitudinal Survey OR Longitudinal Surveys OR Survey, Longitudinal OR Surveys, Longitudinal OR Bogalusa Heart Study OR Bogalusa Heart Studies OR Heart Studies, Bogalusa OR Heart Study, Bogalusa OR Studies, Bogalusa Heart OR Study, Bogalusa Heart OR California Teachers Study OR California Teachers Studies OR  Studies, California Teachers OR Study, California Teachers OR Teachers Studies, California OR Teachers Study, California OR Jackson Heart Study OR Heart Studies, Jackson OR Heart Study, Jackson OR Jackson Heart Studies OR Studies, Jackson Heart OR Tuskegee Syphilis Study OR Syphilis Studies, Tuskegee OR Syphilis Study, Tuskegee OR Tuskegee Syphilis Studies OR Framingham Heart Study OR Framingham Heart Studies OR Heart Studies, Framingham OR Heart Study, Framingham) | 330,628 |
| #5 | #1 AND #2 AND #3 | 312 |

Database: Corchane Library

| Search | Query | Results |
| --- | --- | --- |
| #1 | MeSH descriptor: [Neoplasms] explode all trees | 130,416 |
| #2 | (Tumors):ab,ti,kw OR (Neoplasia):ab,ti,kw OR (Neoplasias):ab,ti,kw OR (Neoplasm):ab,ti,kw OR (Tumor):ab,ti,kw OR (Cancer):ab,ti,kw OR (Cancers):ab,ti,kw OR (Malignant Neoplasm):ab,ti,kw OR (Malignancy):ab,ti,kw OR (Malignancies):ab,ti,kw OR (Malignant Neoplasms):ab,ti,kw OR (Neoplasm, Malignant):ab,ti,kw OR (Neoplasms, Malignant):ab,ti,kw OR (Benign Neoplasms):ab,ti,kw OR (Neoplasms, Benign):ab,ti,kw OR (Neoplasm, Benign):ab,ti,kw OR (Benign Neoplasm):ab,ti,kw | 275,836 |
| **#3** | #1 OR #2 | 300,625 |
| #4 | MeSH descriptor: [Financial Stress] explode all trees | 52 |
| #5 | (Financial Stresses):ab,ti,kw OR (Stress, Financial):ab,ti,kw OR (Economic Burden):ab,ti,kw OR (Burden, Economic):ab,ti,kw OR (Economic Burdens):ab,ti,kw OR (Financial Burden):ab,ti,kw OR (Burden, Financial):ab,ti,kw OR (Financial Burdens):ab,ti,kw OR (Financial Toxicity):ab,ti,kw OR (Financial Toxicities):ab,ti,kw OR (Toxicity, Financial):ab,ti,kw OR (Financial Challenges):ab,ti,kw OR (Challenge, Financial):ab,ti,kw OR (Financial Challenge):ab,ti,kw OR (Financial Pressures):ab,ti,kw OR (Financial Pressure):ab,ti,kw OR (Pressure, Financial):ab,ti,kw OR (Financial Strain):ab,ti,kw OR (Financial Strains):ab,ti,kw OR (Strain, Financial):ab,ti,kw OR (Insufficient Financial Resources):ab,ti,kw OR (Financial Resource, Insufficient):ab,ti,kw OR (Insufficient Financial Resource):ab,ti,kw OR (Resource, Insufficient Financial):ab,ti,kw OR (Financial Resource Strain):ab,ti,kw OR (Financial Resource Strains):ab,ti,kw OR (Resource Strain, Financial):ab,ti,kw OR (Strain, Financial Resource):ab,ti,kw OR (Socioeconomic Adversity):ab,ti,kw OR (Adversity, Socioeconomic):ab,ti,kw OR (Socioeconomic Adversities):ab,ti,kw OR (Financial Hardship):ab,ti,kw OR (Financial Hardships):ab,ti,kw OR (Hardship, Financial):ab,ti,kw OR (Economic Hardship):ab,ti,kw OR (Economic Hardships):ab,ti,kw OR (Hardship, Economic):ab,ti,kw | 7438 |
| **#6** | #4 OR #5 | 7438 |
| #7 | MeSH descriptor: [Longitudinal Studies] explode all trees | 8982 |
| #8 | ('Longitudinal Study':ab,ti,kw OR 'Studies, Longitudinal':ab,ti,kw OR 'Study, Longitudinal':ab,ti,kw OR 'Longitudinal Survey':ab,ti,kw OR 'Longitudinal Surveys':ab,ti,kw OR 'Survey, Longitudinal':ab,ti,kw OR 'Surveys, Longitudinal':ab,ti,kw OR 'Bogalusa Heart Study':ab,ti,kw OR 'Bogalusa Heart Studies':ab,ti,kw OR 'Heart Studies, Bogalusa':ab,ti,kw OR 'Heart Study, Bogalusa':ab,ti,kw OR 'Studies, Bogalusa Heart':ab,ti,kw OR 'Study, Bogalusa Heart':ab,ti,kw OR 'California Teachers Study':ab,ti,kw OR 'California Teachers Studies':ab,ti,kw OR 'Studies, California Teachers':ab,ti,kw OR 'Study, California Teachers':ab,ti,kw OR 'Teachers Studies, California':ab,ti,kw OR 'Teachers Study, California':ab,ti,kw OR 'Jackson Heart Study':ab,ti,kw OR 'Heart Studies, Jackson':ab,ti,kw OR 'Heart Study, Jackson':ab,ti,kw OR 'Jackson Heart Studies':ab,ti,kw OR 'Studies, Jackson Heart':ab,ti,kw OR 'Tuskegee Syphilis Study':ab,ti,kw OR 'Syphilis Studies, Tuskegee':ab,ti,kw OR 'Syphilis Study, Tuskegee':ab,ti,kw OR 'Tuskegee Syphilis Studies':ab,ti,kw OR 'Framingham Heart Study':ab,ti,kw OR 'Framingham Heart Studies':ab,ti,kw OR 'Heart Studies, Framingham':ab,ti,kw OR 'Heart Study, Framingham':ab,ti,kw) | 29,828 |
| **#9** | #7 OR #8 | 29,828 |
| #10 | #3 AND #6 AND #9 | 56 |

Database: Embase

| Search | Query | Results |
| --- | --- | --- |
| #1 | 'neoplasm'/exp | 6,929,051 |
| #2 | tumors:ab,ti,kw OR neoplasia:ab,ti,kw OR neoplasias:ab,ti,kw OR neoplasm:ab,ti,kw OR tumor:ab,ti,kw OR cancer:ab,ti,kw OR cancers:ab,ti,kw OR (malignant:ab,ti,kw AND neoplasm:ab,ti,kw) OR malignancy:ab,ti,kw OR malignancies:ab,ti,kw OR (malignant:ab,ti,kw AND neoplasms:ab,ti,kw) OR (neoplasm,:ab,ti,kw AND malignant:ab,ti,kw) OR (neoplasms,:ab,ti,kw AND malignant:ab,ti,kw) OR (benign:ab,ti,kw AND neoplasms:ab,ti,kw) OR (neoplasms,:ab,ti,kw AND benign:ab,ti,kw) OR (neoplasm,:ab,ti,kw AND benign:ab,ti,kw) OR (benign:ab,ti,kw AND neoplasm:ab,ti,kw) | 5,590,278 |
| **#3** | #2 OR #3 | 7,966,833 |
| #4 | 'financial stress'/exp | 8,974 |
| #5 | (Financial Stresses):ab,ti,kw OR (Stress, Financial):ab,ti,kw OR (Economic Burden):ab,ti,kw OR (Burden, Economic):ab,ti,kw OR (Economic Burdens):ab,ti,kw OR (Financial Burden):ab,ti,kw OR (Burden, Financial):ab,ti,kw OR (Financial Burdens):ab,ti,kw OR (Financial Toxicity):ab,ti,kw OR (Financial Toxicities):ab,ti,kw OR (Toxicity, Financial):ab,ti,kw OR (Financial Challenges):ab,ti,kw OR (Challenge, Financial):ab,ti,kw OR (Financial Challenge):ab,ti,kw OR (Financial Pressures):ab,ti,kw OR (Financial Pressure):ab,ti,kw OR (Pressure, Financial):ab,ti,kw OR (Financial Strain):ab,ti,kw OR (Financial Strains):ab,ti,kw OR (Strain, Financial):ab,ti,kw OR (Insufficient Financial Resources):ab,ti,kw OR (Financial Resource, Insufficient):ab,ti,kw OR (Insufficient Financial Resource):ab,ti,kw OR (Resource, Insufficient Financial):ab,ti,kw OR (Financial Resource Strain):ab,ti,kw OR (Financial Resource Strains):ab,ti,kw OR (Resource Strain, Financial):ab,ti,kw OR (Strain, Financial Resource):ab,ti,kw OR (Socioeconomic Adversity):ab,ti,kw OR (Adversity, Socioeconomic):ab,ti,kw OR (Socioeconomic Adversities):ab,ti,kw OR (Financial Hardship):ab,ti,kw OR (Financial Hardships):ab,ti,kw OR (Hardship, Financial):ab,ti,kw OR (Economic Hardship):ab,ti,kw OR (Economic Hardships):ab,ti,kw OR (Hardship, Economic):ab,ti,kw | 134,695 |
| **#6** | #4 OR #5 | 137,768 |
| #7 | 'longitudinal study'/exp | 262,220 |
| #8 | ('Longitudinal Study':ab,ti,kw OR 'Studies, Longitudinal':ab,ti,kw OR 'Study, Longitudinal':ab,ti,kw OR 'Longitudinal Survey':ab,ti,kw OR 'Longitudinal Surveys':ab,ti,kw OR 'Survey, Longitudinal':ab,ti,kw OR 'Surveys, Longitudinal':ab,ti,kw OR 'Bogalusa Heart Study':ab,ti,kw OR 'Bogalusa Heart Studies':ab,ti,kw OR 'Heart Studies, Bogalusa':ab,ti,kw OR 'Heart Study, Bogalusa':ab,ti,kw OR 'Studies, Bogalusa Heart':ab,ti,kw OR 'Study, Bogalusa Heart':ab,ti,kw OR 'California Teachers Study':ab,ti,kw OR 'California Teachers Studies':ab,ti,kw OR 'Studies, California Teachers':ab,ti,kw OR 'Study, California Teachers':ab,ti,kw OR 'Teachers Studies, California':ab,ti,kw OR 'Teachers Study, California':ab,ti,kw OR 'Jackson Heart Study':ab,ti,kw OR 'Heart Studies, Jackson':ab,ti,kw OR 'Heart Study, Jackson':ab,ti,kw OR 'Jackson Heart Studies':ab,ti,kw OR 'Studies, Jackson Heart':ab,ti,kw OR 'Tuskegee Syphilis Study':ab,ti,kw OR 'Syphilis Studies, Tuskegee':ab,ti,kw OR 'Syphilis Study, Tuskegee':ab,ti,kw OR 'Tuskegee Syphilis Studies':ab,ti,kw OR 'Framingham Heart Study':ab,ti,kw OR 'Framingham Heart Studies':ab,ti,kw OR 'Heart Studies, Framingham':ab,ti,kw OR 'Heart Study, Framingham':ab,ti,kw) | 132,232 |
| **#9** | #7 OR #8 | 296,461 |
| #10 | #3 AND #6 AND #9 | 419 |
