# Supplementary material for: Longitudinal studies on financial toxicity in cancer patients: a scoping review
Source: Front Public Health. 2026 Mar 11;14:1798871. doi: 10.3389/fpubh.2026.1798871 (PMC13013514; doi:10.3389/fpubh.2026.1798871)
Supplement: Supplementary file 2 [file Table_2.DOCX]

Date：2025-11-08

Database：CKNI

| Search | Query | Results |
| --- | --- | --- |
| #1 | SU=肿瘤 + 癌症 + 恶性肿瘤 + 肿瘤，恶性 AND SU=经济毒性 + 财务压力 + 压力，财务 + 经济负担 + 负担，经济 + 财政负担 + 财务负担 + 金融毒性 + 毒性，金融 + 财务挑战 + 挑战，金融 + 财务挑战 + 经济压力 + 财政压力 + 资金紧张 + 财务压力 + 财务资源不足 + 资源，资金不足 + 财务资源紧张 + 财政资源紧张 + 资源紧张，财务 + 压力、财务资源 + 社会经济逆境 + 逆境，社会经济 + 社会经济逆境 + 财务困难 + 财政困难 + 艰难，财务 + 经济困难 + 艰难，经济 AND SU=纵向研究 + 队列研究 + 随访研究 + 前瞻性研究 + 追踪研究 + 长期随访 | 21 |

Database：Wan Fang

| Search | Query | Results |
| --- | --- | --- |
| #1 | 题名或关键词:(肿瘤 OR 癌症 OR 恶性肿瘤 OR 肿瘤，恶性 ) and 题名或关键词:(经济毒性 OR 财务压力 OR 压力，财务 OR 经济负担 OR 负担，经济 OR 财政负担 OR 财务负担 OR 金融毒性 OR 毒性，金融 OR 财务挑战 OR 挑战，金融 OR 财务挑战 OR 经济压力 OR 财政压力 OR 资金紧张 OR 财务压力 OR 财务资源不足 OR 资源，资金不足 OR 财务资源紧张 OR 财政资源紧张 OR 资源紧张，财务 OR 压力、财务资源 OR 社会经济逆境 OR 逆境，社会经济 OR 社会经济逆境 OR 财务困难 OR 财政困难 OR 艰难，财务 OR 经济困难 OR 艰难，经济) and 题名或关键词:(纵向研究 OR 队列研究 OR 随访研究 OR 前瞻性研究 OR 追踪研究 OR 长期随访) | 16 |

Database：VIP

| Search | Query | Results |
| --- | --- | --- |
| #1 | (U=肿瘤 OR 癌症 OR 恶性肿瘤 OR 肿瘤，恶性) AND (U=经济毒性 OR 财务压力 OR 压力，财务 OR 经济负担 OR 负担，经济 OR 财政负担 OR 财务负担 OR 金融毒性 OR 毒性，金融 OR 财务挑战 OR 挑战，金融 OR 财务挑战 OR 经济压力 OR 财政压力 OR 资金紧张 OR 财务压力 OR 财务资源不足 OR 资源，资金不足 OR 财务资源紧张 OR 财政资源紧张 OR 资源紧张，财务 OR 压力、财务资源 OR 社会经济逆境 OR 逆境，社会经济 OR 社会经济逆境 OR 财务困难 OR 财政困难 OR 艰难，财务 OR 经济困难 OR 艰难，经济) AND (U=纵向研究 OR 队列研究 OR 随访研究 OR 前瞻性研究 OR 追踪研究 OR 长期随访) | 81 |

Database: Sinomed（CBM）

| Search | Query | Results |
| --- | --- | --- |
| #1 | (("癌症"[常用字段:智能] OR "恶性肿瘤"[常用字段:智能] OR "肿瘤，恶性"[常用字段:智能] ) OR ("肿瘤"[不加权:扩展])) AND ("影响因素"[常用字段:智能] OR "危险因素"[常用字段:智能] OR "病因"[常用字段:智能] OR "相关因素"[常用字段:智能]) AND ("经济毒性"[常用字段:智能] OR "财务压力"[常用字段:智能] OR "压力，财务"[常用字段:智能] OR "经济负担"[常用字段:智能] OR "负担，经济"[常用字段:智能] OR "财政负担"[常用字段:智能] OR "财务负担"[常用字段:智能] OR "金融毒性"[常用字段:智能] OR "毒性，金融"[常用字段:智能] OR "财务挑战"[常用字段:智能] OR "挑战，金融"[常用字段:智能] OR "财务挑战"[常用字段:智能] OR "经济压力"[常用字段:智能] OR "财政压力"[常用字段:智能] OR "资金紧张"[常用字段:智能] OR "财务压力"[常用字段:智能] OR "财务资源不足"[常用字段:智能] OR "资源，资金不足"[常用字段:智能] OR "财务资源紧张"[常用字段:智能] OR "财政资源紧张"[常用字段:智能] OR "资源紧张，财务"[常用字段:智能] OR "压力、财务资源"[常用字段:智能] OR "社会经济逆境"[常用字段:智能] OR "逆境，社会经济"[常用字段:智能] OR "社会经济逆境"[常用字段:智能] OR "财务困难"[常用字段:智能] OR "财政困难"[常用字段:智能] OR "艰难，财务"[常用字段:智能] OR "经济困难"[常用字段:智能] OR "艰难，经济"[常用字段:智能]) AND ( "纵向研究"[常用字段:智能] OR "队列研究"[常用字段:智能] OR "随访研究"[常用字段:智能] OR "前瞻性研究"[常用字段:智能] OR "追踪研究"[常用字段:智能] OR "长期随访"[常用字段:智能] ) | 28 |
